# Tailoring the Microstructure of a Solid Oxide Fuel Cell Anode Support by Calcination and Milling of YSZ

**DOI:** 10.1038/srep27359

**Published:** 2016-06-07

**Authors:** Amir Reza Hanifi, Miguel A. Laguna-Bercero, Navjot Kaur Sandhu, Thomas H. Etsell, Partha Sarkar

**Affiliations:** 1 Department of Chemical & Materials Engineering, University of Alberta, Edmonton, Alberta T6G 1H9, Canada; 2 Instituto de Ciencia de Materiales de Aragón (ICMA), CSIC- Universidad de Zaragoza, C/Pedro Cerbuna 12, E-50009, Zaragoza, Spain; 3 Environment & Carbon Management, Alberta Innovates - Technology Futures, Edmonton, Alberta. T6N 1E4, Canada

## Abstract

In this study, the effects of calcination and milling of 8YSZ (8 mol% yttria stabilized zirconia) used in the nickel-YSZ anode on the performance of anode supported tubular fuel cells were investigated. For this purpose, two different types of cells were prepared based on a Ni-YSZ/YSZ/Nd_2_NiO_4+δ_-YSZ configuration. For the anode preparation, a suspension was prepared by mixing NiO and YSZ in a ratio of 65:35 wt% (Ni:YSZ 50:50 vol.%) with 30 vol.% graphite as the pore former. As received Tosoh YSZ or its calcined form (heated at 1500 °C for 3 hours) was used in the anode support as the YSZ source. Electrochemical results showed that optimization of the fuel electrode microstructure is essential for the optimal distribution of gas within the support of the cell, especially under electrolysis operation where the performance for an optimized cell (calcined YSZ) was enhanced by a factor of two. In comparison with a standard cell (containing as received YSZ), at 1.5 V and 800 °C the measured current density was −1380 mA cm^−2^ and −690 mA cm^−2^ for the cells containing calcined and as received YSZ, respectively. The present study suggests that the anode porosity for improved cell performance under SOEC is more critical than SOFC mode due to more complex gas diffusion under electrolysis mode where large amount of steam needs to be transfered into the cell.

Nickel-YSZ is the commonly used anode material in solid oxide fuel cells (SOFCs) due to its high performance at intermediate temperatures. The fuel cell anode needs to have a suitable microstructure resulting in high electronic conductivity as well as low activation and concentration polarizations. In order to obtain high electrical conductivity, nickel particles need to form a percolative network. Nickel also provides high catalytic activity and transfers the electrons from the functional layer to the current collector. Sufficient anode porosity is crucial for the fuel gas to diffuse and for the removal of the reaction products. However, porosity needs to be optimized without negatively affecting the mechanical strength of the anode^1^,^2^.

YSZ has several functions in the anode microstructure including providing ionic conductivity, limiting nickel sintering, matching the thermal expansion coefficient of the anode with the electrolyte and broadening the triple phase boundaries (TPBs), where nickel (electronic conductor), YSZ (ionic conductor) and pores (fuel gas channels) meet^3^,^4^,^5^. Electrochemical performance and durability of the anode is a function of microstructure and thus TPB length. Larger TPB length leads to a reduction in polarization^4^. It is believed that a finer microstructure having a uniform distribution of particles and pores increases the TPB length^5^,^6^.

The effect of nickel particle size and distribution on the overall anode performance has been studied by several researchers where it was found that the anode conductivity is highly affected by the powder synthesis technique and was shown that an anode with finer microstructure provides lower resistance^7^,^8^,^9^. The Ni-YSZ ratio has also been identified as an important criterion in the extension of the TPBs. High nickel or YSZ content has an adverse effect on the TPB length^9^. Wilson *et al*. identified the highest TPB length when nickel was 34% of the solid volume^8^. Of particular interest is the distribution of channels along the fuel electrode, especially under solid oxide electrolysis cell (SOEC) mode, as high amounts of steam must be transported^9^. Anode porosity has been found to be an important factor in cell microstructure and thus power performance. Porosities in the range of 50 vol.% for the anode supported microtubular cells are typically used^10^. Suzuki *et al*.^11^ varied the anode porosity in anode supported SOFCs by controlling the sintering temperature and found that the more porous sample (54%) had finer nickel particles and better performance due to the ease of gas diffusion. The effect of gas diffusion using infiltrated electrodes was studied in detail by Hussain *et al*.^12^. They found that the resulting impedance due to gas diffusion in infiltrated electrodes showed a clear dependency on the structural parameters of the electrode. The effect of gas diffusion is even more important in electrolysis mode. Recently, Ebbesen *et al*.^13^ studied this effect in Ni-YSZ anode supported cells with different porosity (28% and 34%). They found that changing the porosity of the support structure results in a change in the Ni–YSZ TPB resistance and a significant change in the low frequency concentration related resistance at high current densities. They observed increased polarization in EC-mode while decreasing the porosity shows that diffusion limitations cannot be neglected for support structures with porosities below 30% (with a support thickness of 300 μm).

The effectiveness of using calcined YSZ in the development of a porous structure for infiltration of nickel^14^,^15^ or LSBT^16^ anodes as well as LSM^17^,^18^ or Nd nickelate^19^ cathodes has been previously shown by the authors. Despite many studies on the effect of nickel particle size and content on anode microstructure and thus performance, there is a lack of information in the literature regarding the effect of using processed YSZ in the conventional nickel-YSZ anode on electrochemical performance. The aim of the current study is to investigate the influence of using calcined-milled YSZ vs. as received YSZ in the anode microstructure on cell performance under both SOFC and SOEC modes. In this manuscript the anode supported fuel cells with as received Tosoh YSZ and calcined YSZ in their Ni-YSZ anode support are referred to as “TY” and “CY”, respectively.

## Results and Discussion

### Microstructural analysis

**Figure 1:**
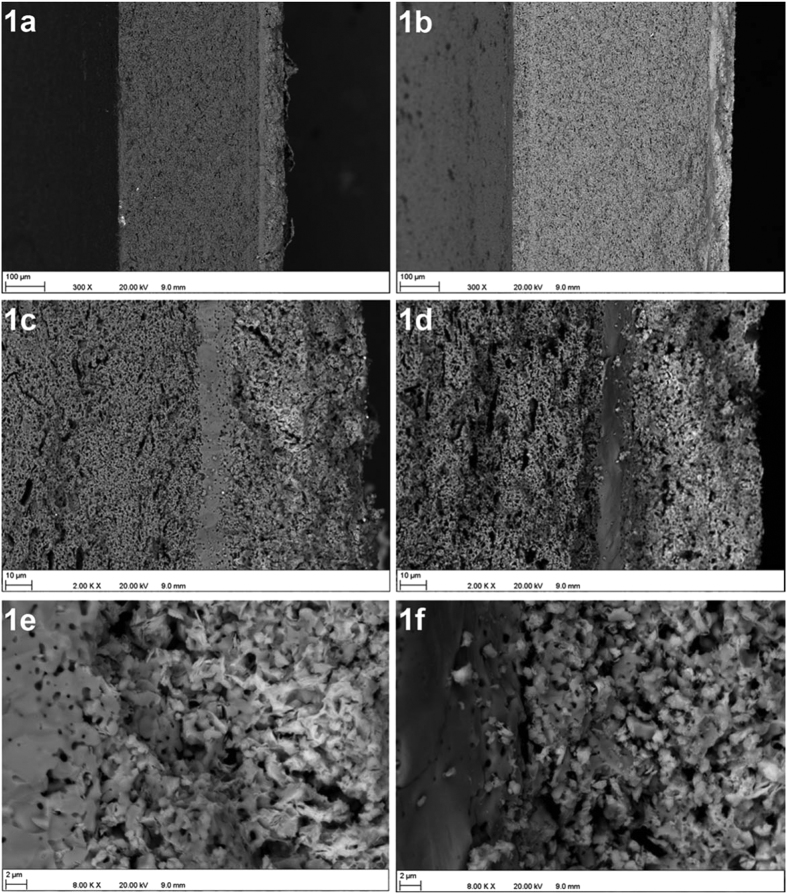
SEM image showing the microstructure of cell TY (**a,c,e**) and cell CY (**b,d,f**). (**a**) Cross-section of cell TY, (**b**) Cross-section of cell CY, (**c**) Interfaces between cathode (right), electrolyte and the anode (left) in cell TY, (**d**) Interfaces between cathode (right), electrolyte and the anode (left) in cell CY, (**e**) YSZ coverage by Nd nickelate at the interface of cathode and electrolyte in cell TY and (**f**) YSZ coverage by Nd nickelate at the interface of cathode and electrolyte in cell CY.

Figure 1a,b represent the cross-sections of TY and CY following electrochemical testing. The thicknesses of the anode supports are about 360 and 500 μm for the TY and CY cells, respectively. The interfaces between the entire cathode thickness, electrolyte and part of the anode for the TY and CY cells are shown in Fig. 1c,d, respectively. The cathode and the electrolyte shown for both cells have similar thicknesses of about 50 μm and 13 μm, respectively. The anode microstructure shows the pores formed due to the burning of the graphite pore-former (slit shaped pores), and smaller pores which correspond to intergranular pores and porosity formed due to the reduction of NiO to Ni. It is worth noting that the anode of the TY cell is less porous with finer pores compared with the anode of the CY cell. Figure 1e,f shows a suitable distribution of the Nd nickelate cathode (needles or plate like particles) near the interface of the cathode and electrolyte. Considering the weight gain of the cathode following infiltration of Nd nicklate, the YSZ:Nd nickelate ratio was calculated to be 69:31 vol.% and a 14% decrease in the total open porosity of the cathode was found leading to a porosity reduction from 50% to 36%.

**Figure 2:**
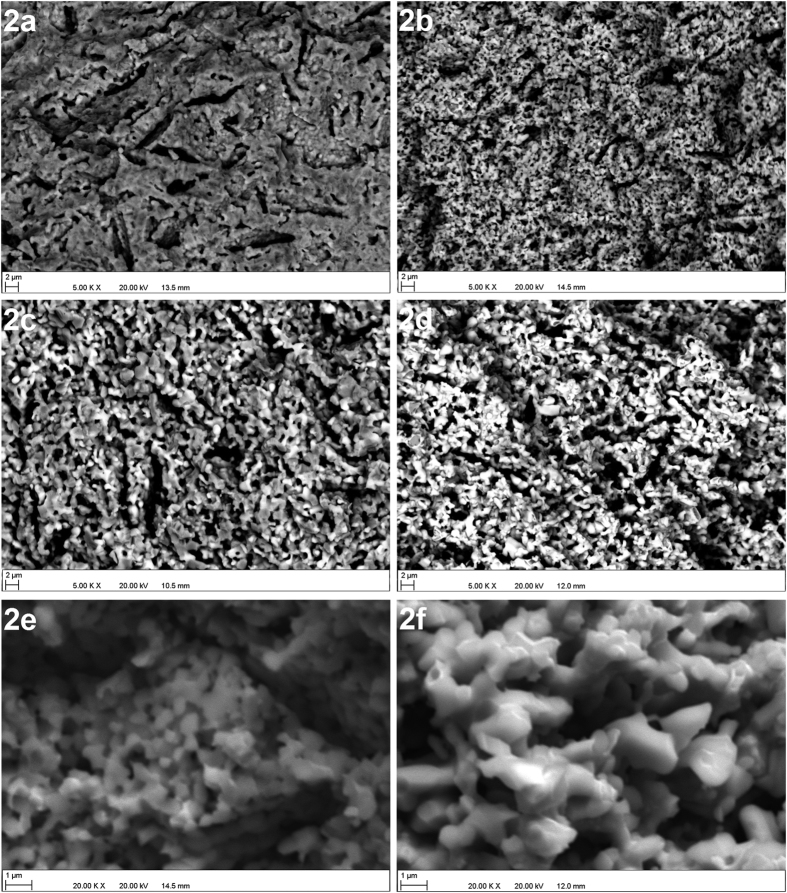
SEM image of (**a**) TY anode before reduction, (**b**) TY anode after reduction, (**c**) CY anode before reduction, (**d**) CY anode after reduction, (**e**) Pores of TY anode, (**f**) Pores of CY anode.

Table 1 shows the porosity of the TY and CY anodes before and after reduction. Following sintering at 1350 °C, both microstructures have a significant amount of closed pores (see Fig. 2a,c). After reduction, the amount of closed porosity in both microstructures decreases and open porosity increases (see Fig. 2b,d). The CY anode microstructure remains more porous than the TY anode both before and after reduction. The pores caused by the pore former are larger (5–20 μm) than the intergranular pores and the porosity caused by NiO reduction (∼1 μm). These finer pores have more impact on the triple phase boundary length than the larger pores formed by graphite. However, larger pores provide excellent channels for gas diffusion into the reaction points. The comparison of the high magnification images shown in Fig. 2e,f reveals that the less porous TY anode contains finer pores. The distribution of the pores (not formed by the graphite pore-former) and their average size in both anodes presented in Table 1 confirms this. This might be the reason for its similar surface area (see Table 1) to the CY anode despite the fact that the former anode is less porous.

It was previously shown by the authors that following 72 hr ball milling of as received Tosoh YSZ, its particle size (250 nm for as received powder and 240 nm following milling) and surface area (13.19 m^2^/g for as received powder and 12.38 m^2^/g following milling) remain relatively constant^20^. However, the particle size of 1500 °C calcined YSZ (75 μm) shows a significant decrease following 72 hr ball milling (760 nm) and its surface area after calcining (0.03 m^2^/g) increases following milling (3.23 m^2^/g). Therefore, the milled-calcined YSZ maintains larger particles and a lower surface area compared with the as received powder. The increased particle size of calcined-milled YSZ compared with as received powder is a major reason for the reduced sinterability high porosity and larger pores in the anode microstructures containing calcined powder. This is also confirmed by the lower shrinkage rate of this sample following sintering (see Table 1).

### Electrochemical Characterization

**Figure 3:**
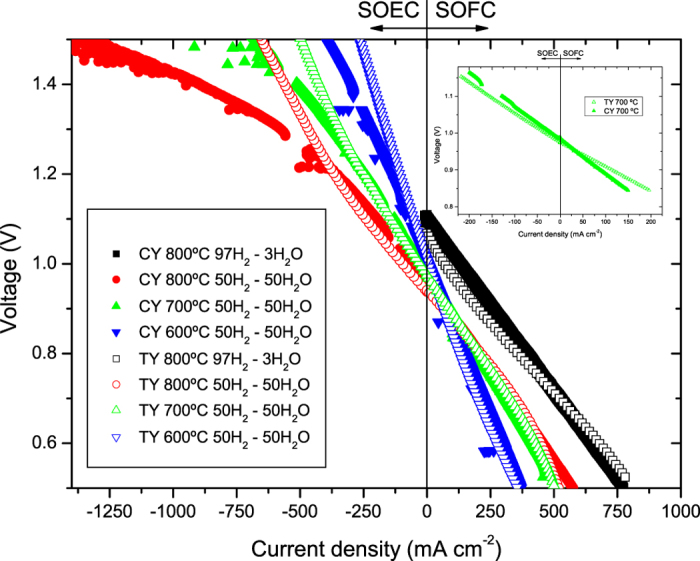
Electrochemical galvanodynamic studies in SOFC and SOEC modes for the TY and CY cells. Solid symbols correspond to the CY cell and hollow symbols to the TY cell. Black: measured at 800 °C using RT humidified hydrogen as fuel. All the rest were measured using 50% steam −50% hydrogen as fuel. Red: measured at 800 °C; Green: measured at 700 °C; Blue: measured at 600 °C. The inset corresponds to a magnification for both samples at low current densities (700 °C).

The electrochemical performance (current density versus voltage curves) for TY and CY cells under fuel cell and electrolysis operation modes can be observed in Fig. 3. Initial characterization was performed at 800 °C under pure humidified hydrogen (100 mL/min H_2_ through a water bubbler kept at room temperature: ~3 vol.% steam content). As observed in the figure (black solid and hollow squared symbols), the performance of both TY and CY cells is rather similar under SOFC mode, as current densities of about 600 mA cm^−2^ were achieved for both cells at 0.7 V. However, this value is much higher than that obtained for cells fabricated using the same methodology but using standard LSM-YSZ oxygen electrodes, where current densities in the range of 380 mA cm^−2^ were measured under identical operating conditions^21^, and also higher than standard anode supported micro-tubular cells (NiO-YSZ/YSZ/LSM-YSZ)^10^. In addition, comparable values were reported by the authors for similar cells also using Nd nickelate infiltrated into a thin porous YSZ layer as the cathode^19^.

**Figure 4:**
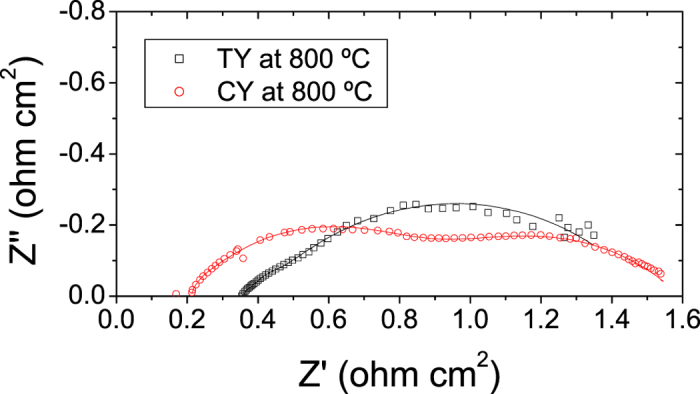
EIS experiments recorded at OCV conditions and 800 °C for the TY and CY cells. Symbols correspond to experimental data and solid lines correspond to the fitting.

EIS spectra recorded under OCV conditions are plotted in Fig. 4, and the fitted parameters, using the *LR*_s_(*R*_1_/CPE_1_)(*R*_*2*_/CPE_2_)(*R*_3_/CPE_3_) equivalent circuit, are summarized in Table 2. Although ASR values are reasonably similar for both cells (1.48 Ωcm^2^ and 1.55 Ωcm^2^ for the TY and CY cell, respectively), the EIS spectra are rather different. As observed in Table 2, R_s_ is smaller for the CY cell. This is consistent with the densification of the YSZ electrolyte, as some porosity is observed for the TY cell (Fig. 1c). The co-sintering process of the green tube/electrolyte layer for the TY cell could be further optimized. Poor lateral current collection might be also contributing to this increased ohmic resistance for the TY cell. In addition, the R_1_ contribution is in the range of 0.035–0.04 Ωcm^2^ appearing at frequencies of ∼10–20 kHz. As previously observed by different authors, this process is attributed to charge transfer at the oxygen electrode/electrolyte interface^22^,^23^. Furthermore, R_1_, the high frequency (HF) component, is not changing for both cells, confirming that this contribution is produced by the common Nd nickelate-YSZ oxygen electrode. R_2_, the medium frequency (MF) component, and R_3_, the low frequency (LF) component, occurring at frequencies of ∼0.1–2 kHz and ∼1–10 Hz, are generally attributed to charge transfer and gas diffusion at the fuel electrode, respectively^10^,^24^. As observed in Table 2, when using the calcined powder (CY cell), the LF component was lower (improved diffusion in the anode support as a consequence of the optimized porosity) whereas the MF component was higher. The increase of the MF component is due to lower activation energy which is consistent with the larger pore distribution which affects the TPBs. CY has higher activation polarization but lower concentration polarization than TY and this can be the reason for their similar power performance under SOFC mode.

SOFC-SOEC electrochemical characterization was also performed using a high steam concentration (mixtures of 50% steam–50% hydrogen as fuel) (see Fig. 3). Under these conditions, although the performance of both TY and CY cells is rather similar under SOFC mode, concentration polarization is clearly observed at high current densities for the TY cell (above ∼400 mAcm^−2^ at 700 °C and 800 °C), as a consequence of the non-optimized porosity obstructing an appropriate flux of hydrogen and steam. This effect is much more noticeable under electrolysis (SOEC) mode, despite the wall thickness difference of the anode supports (∼360 and ∼500 μm for the TY and CY cells, respectively). Much higher current densities were measured for the CY cell as a consequence of the greater number and larger pores at the fuel electrode, especially when increasing the voltage and the operating temperature. For example, at 1.5 V and 800 °C, the measured current density for the CY cell was increased by a factor of two in comparison with the TY cell (−1380 mA cm^−2^ and −690 mA cm^−2^, respectively). The performance of the TY cell is similar to other micro-tubular SOEC cells reported in the literature^25^. From our knowledge, the SOEC performance of the CY cell is the highest reported for micro-tubular electrolysis cells^26^. The current results confirm that the pore content and its size distribution in the CY cell are responsible for the increased performance, in particular for electrolysis applications.

**Figure 5:**
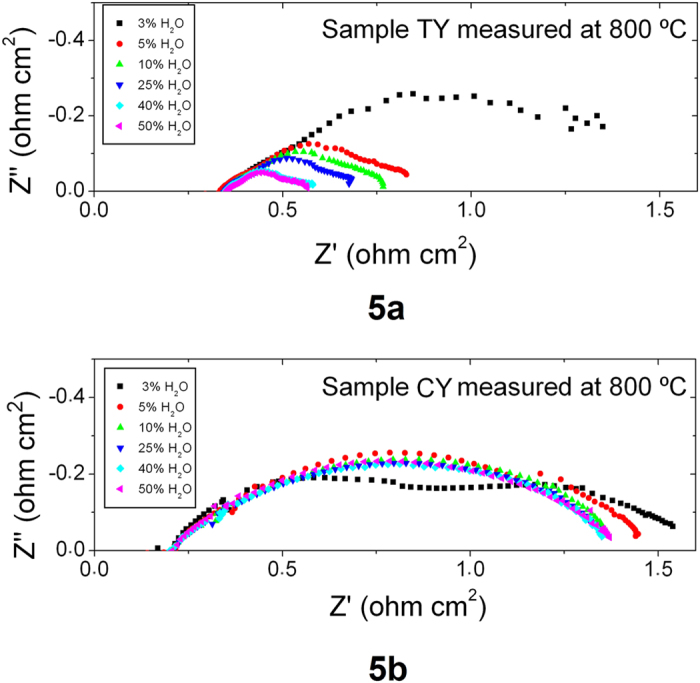
EIS experiments recorded at OCV conditions and 800 °C as a function of *p*H_2_O for the (**a**) TY and (**b**) CY cells.

The effect of gas transport for both cells is illustrated in the EIS experiments performed as a function of the steam content in the fuel electrode (see Fig. 5) under OCV conditions. Using low steam contents for the TY cell, the polarization resistance of the cell significantly increases, as a consequence of a gas transport limitation. When increasing the steam content, this polarization resistance is reduced. However, for the CY sample even for low steam contents, the polarization resistance is approximately constant for the full range studied as a consequence of the optimized porosity.

**Figure 6:**
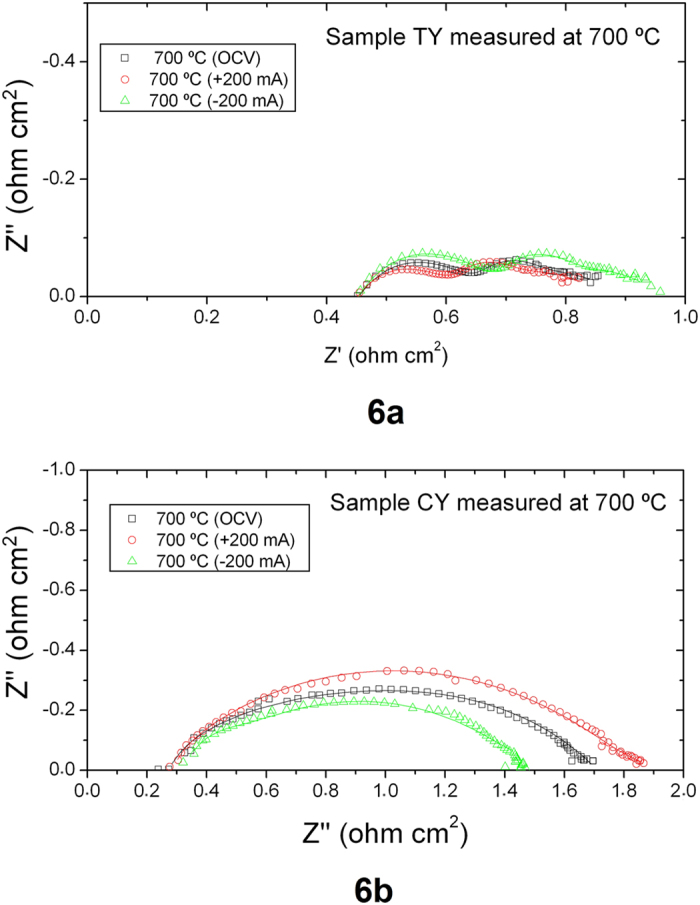
EIS experiments recorded under current load at 700 °C for the (**a**) TY and (**b**) CY cells.

Additional information can be obtained from the EIS data generated under current load (Fig. 6). Analysis of the impedance spectra for single cells using equivalent circuits is complex, as some of the electrode processes usually overlap, as is the case for the CY cell (Fig. 6b). The fitted parameters, using the equivalent circuit previously described, are also summarized in Table 2. It is important to note that these experiments were performed using moderate current loads (±200 mA) due to limitations of the equipment. As observed in the inset of Fig. 3, at those current densities, the TY cell outperforms the CY cell, as in this region of the *j*-V curve activation polarization is more dominant than concentration polarization. In any case, these experiments confirmed that the HF contribution corresponds to the Nd_2_NiO_4+δ_-YSZ oxygen electrode as it remained almost constant for all the conditions studied in both cells. The most significant change is the decrease of the LF contribution for the CY cell under negative polarization (SOEC conditions), as a consequence of the optimized gas diffusion within the Ni-YSZ support. Positive impacts of using calcined YSZ in developing a porous YSZ microstructure for conversion to an anode or cathode upon infiltration has been previously shown by the authors^14^,^15^,^16^,^18^,^19^,^21^,^27^,^28^. When cell infiltration for the purpose of improving the catalytic activity or the electronic/ionic conductivity (or both) of the anode is planned, an anode with larger pore size and higher porosity (~50%) than TY is desirable (due to pore clogging during infiltration). CY can offer the potential microstructure very effectively. It is also noticeable that the initial particle size of both calcined YSZ and NiO following 72 h ball milling is submicron for which, according to Yu *et al*.^29^, the resulting anode cermet has a fine microstructure of well percolating phases which provides suitable electrical and mechanical properties. They have shown that very large particles of NiO and YSZ have negative effects on both the electrical and mechanical properties of the anode cermet.

## Conclusions

From the comparison of the anodes containing as received Tosoh YSZ or calcined YSZ, the following conclusions can be drawn:

1. The anode containing as received Tosoh YSZ had less porosity (33%) compared with the anode containing calcined-milled YSZ (46%). This negatively affects the gas diffusion in the first anode especially under SOEC mode.
2. In the case of the anode containing as received Tosoh YSZ, the pores located in the anode functional layer contributing to the triple phase boundaries are finer which can lead to an increase in TPB length and reduction of the cell activation polarization.
3. The anode containing calcined YSZ shows sufficient porosity for infiltration thereby enhancing its electrochemical performance while the anode containing Ni-Tosoh YSZ can have blocked pores following infiltration.
4. Electrochemical performance in SOFC mode is similar for the as received Tosoh YSZ and the calcined YSZ cell. For the calcined cell, gas diffusion improved while activation polarization deteriorated. These contributions compensated each other.
5. Gas diffusion plays a crucial role in electrolysis mode. An optimized microstructure of the anode support led to an increase by a factor of two of the current density at 800 °C and 1.5 V.

## Methods

### Cell Fabrication

The anode supported cells studied in this paper were fabricated by slip casting of a NiO-YSZ anode support followed by dip coating of a thin YSZ electrolyte and a thin porous YSZ layer for cathode infiltration. Nd_2_NiO_4+δ_ was infiltrated into the thin porous YSZ layer of both cells to form the cathode.

In order to prepare a suitable slip for casting the anode supported cells, as received YSZ (TZ-8Y, 8 mol% Y_2_O_3_, Tosoh) or its calcined form (calcined at 1500 °C for 3 h) was mixed with 65 wt% NiO powder (Baker Chemicals, <3 μm) and water at a powder:water weight ratio of 1:1. The mixture was then milled at 120 rpm for 72 h in a plastic bottle with 5 mm zirconia balls. Additional water was added after milling to adjust the solid loading of the final suspension to 40%. The pH of the slip was set to 4.0 using 2% hydrochloric acid. In order to generate high porosity, 30 vol.% graphite (Sigma Aldrich <325 mesh) was incorporated into the slip following pH adjustment, and then the suspension was mixed for 15 minutes prior to slip casting.

To create the tubular support, the slip was cast into a plaster mold (previously prepared from a tubular mandrel) and left for about 1 minute, after which the excess slip was quickly poured out. Several pellets (15 mm diameter, 5 mm thick) were also slip cast in a plaster mold using the same slip. The wet tube and pellets were then dried at room temperature for 1 h. The resulting drying shrinkage facilitates removal of the green bodies. The green tube was dried at 100 °C, heated at 700 °C for 1 h to oxidize all the graphite, and then pre-sintered under air at 1100 °C for 3 h. The slip cast pellets were also sintered under a similar sintering regime except their final sintering temperature was 1350 °C.

The electrolyte and the thin porous YSZ layer formulae and their dip coating procedure are explained elsewhere^14^,^15^,^28^. Both layers were sintered at 1350 °C for 3 h. Infiltration of Nd_2_NiO_4+δ_ into the thin porous YSZ layer has also been addressed^19^. Density and porosity measurements were carried out on the slip cast and sintered pellets using Archimedes principle. The same pellets were used for calculation of the sintering shrinkage.

### Characterization

Krypton adsorption/desorption isotherms at 77°K and surface area measurements on the reduced anode pellets were performed by Quantachrome Autosorb-1. Scanning electron microscopy (SEM) was carried out on the fuel cells and anode pellets using a Zeiss EVO LS15 EP-SEM instrument. The pore size of the reduced anode samples was measured by SEM image analysis.

### Electrochemical measurements

Electrochemical studies were performed in the temperature range between 600 °C and 800 °C in both fuel cell and electrolysis modes using a similar experimental setup as previously described^18^,^19^,^21^. A fuel composition of 97% H_2_–3% H_2_O was used for operation in fuel cell mode, and 50% H_2_O–50% H_2_ was used for operation in reversible mode.

For the inner contact (hydrogen electrode), silver wires were welded onto silver mesh and mechanically pressed inside the micro-tubes (6mm inner diameter, 60 mm long). Silver paste was also added to the mesh to improve contact. For the outer contact (oxygen electrode), a thin gold layer was added by dip coating and, subsequently, a gold wire was coiled around the cathode surface (1 cm^2^) and Au paste was added to improve electrical contact and current collection. The cells were then sealed to an alumina tube using an alumina-based ceramic sealant (Aremco, Ceramabond 503) and heated to 800 °C under nitrogen, while the oxygen electrode side was exposed to ambient air. Subsequently, nitrogen gas was switched to pure humidified hydrogen, reducing NiO to metallic Ni at the anode. Steam was supplied by the use of a direct vapour humidifier controlling the relative humidity with a resolution of ±1.3%. All gas lines located downstream of the humidifier were externally heated in order to prevent steam condensation.

*j*-V (current density-voltage) was recorded in galvanodynamic mode using a scan rate of 2.5 mA cm^–2^ s^–1^. EIS (electrochemical impedance spectroscopy) measurements were performed under OCV (open circuit voltage) conditions and also under current load (±200 mA), using 20 mV of sinusoidal amplitude and a frequency range from 100 kHz to 100 mHz. These experiments were performed using a VSP potentiostat/galvanostat (Princeton Applied Research, Oak Ridge, USA).

## Additional Information

**How to cite this article**: Hanifi, A. R. *et al*. Tailoring the Microstructure of a Solid Oxide Fuel Cell Anode Support by Calcination and Milling of YSZ. *Sci. Rep.* **6**, 27359; doi: 10.1038/srep27359 (2016).

## Figures and Tables

**Table 1:** Porosity, surface area, pore size and shrinkage of the TY and CY anodes before and after reduction.

Sample	Open porosity (%)	Closed porosity (%)	Surface area (m^2^/g)	Pore size range (μm)	Average pore size (μm)	Shrinkage (%)
TY before reduction	8 ± 1	18 ± 0.5	–	–	–	20.3
TY after reduction	33 ± 1	3 ± 0.5	0.89	0.3–1.85	0.86	–
CY before reduction	33 ± 1	11 ± 0.5	–	–	–	15.7
CY after reduction	46 ± 1	3 ± 0.5	0.71	0.3–3	1.25	–

**Table 2:** Summary of the fitted parameters from EIS data shown in Figs 4 and 6 under different operating conditions for the TY and CY cells.

Sample	Temp (°C)	Steam content (%)	Polarization	R_s_ (Ωcm^2^)	R_1_-HF (Ωcm^2^) ∼10–20 kHz	R_2_-MF (Ωcm^2^) ∼0.1–2 kHz	R_3_-LF (Ωcm^2^) ∼1–10 Hz	ASR (Ωcm^2^)
TY	800	3	OCV	0.31 ± 0.02	0.035 ± 0.004	0.036 ± 0.005	1.10 ± 0.05	1.48 ± 0.08
TY	700	50	OCV	0.44 ± 0.01	0.27 ± 0.01	0.043 ± 0.002	0.11 ± 0.01	0.86 ± 0.03
TY	700	50	200 mA	0.42 ± 0.03	0.26 ± 0.02	0.041 ± 0.002	0.11 ± 0.01	0.83 ± 0.06
TY	700	50	−200 mA	0.47 ± 0.03	0.26 ± 0.02	0.057 ± 0.002	0.14 ± 0.01	0.93 ± 0.06
CY	800	3	OCV	0.19 ± 0.01	0.04 ± 0.01	0.58 ± 0.02	0.74 ± 0.02	1.55 ± 0.06
CY	700	50	OCV	0.21 ± 0.01	0.22 ± 0.01	0.54 ± 0.01	0.68 ± 0.02	1.65 ± 0.05
CY	700	50	200 mA	0.21 ± 0.01	0.20 ± 0.02	0.81 ± 0.02	0.62 ± 0.02	1.84 ± 0.07
CY	700	50	−200 mA	0.22 ± 0.01	0.20 ± 0.02	0.86 ± 0.03	0.20 ± 0.02	1.48 ± 0.08

Standard errors obtained from the fittings are also shown.
